# Human Serum Amyloid a Impaired Structural Stability of High-Density Lipoproteins (HDL) and Apolipoprotein (Apo) A-I and Exacerbated Glycation Susceptibility of ApoA-I and HDL

**DOI:** 10.3390/molecules27134255

**Published:** 2022-07-01

**Authors:** Kyung-Hyun Cho

**Affiliations:** 1 LipoLab, Yeungnam University, Gyeongsan 38541, Korea; chok@yu.ac.kr; Tel.: +82-53-964-1990; Fax: +82-53-965-1992; 2 Raydel Research Institute, Medical Innovation Complex, Daegu 41061, Korea

**Keywords:** human serum amyloid A, apolipoprotein A-I, reconstituted high-density lipoprotein, oxidation, glycation

## Abstract

Human serum amyloid A (SAA) is an exchangeable apolipoprotein (apo) in high-density lipoprotein (HDL) that influences HDL quality and functionality, particularly in the acute phase of inflammation. On the other hand, the structural and functional correlations of HDL containing SAA and apoA-I have not been reported. The current study was designed to compare the change in HDL quality with increasing SAA content in the lipid-free and lipid-bound states in reconstituted HDL (rHDL). The expressed recombinant human SAA1 (13 kDa) was purified to at least 98% and characterized in the lipid-free and lipid-bound states with apoA-I. The dimyristoyl phosphatidylcholine (DMPC) binding ability of apoA-I was impaired severely by the addition of SAA, while SAA alone could not bind with DMPC. The recombinant human SAA1 was incorporated into the rHDL (molar ratio 95:5:1, 1-palmitoyl-2-oleoyl-sn-glycero-3-phosphocholine (POPC): cholesterol: apoA-I) with various apoA-I:SAA molar ratios from 1:0 to 1:0.5, 1:1 and 1:2. With increasing SAA1 content, the rHDL particle size was reduced from 98 Å to 93 Å, and the α-helicity of apoA-I:SAA was decreased from 73% to 40% for (1:0) and (1:2), respectively. The wavelength maximum fluorescence (WMF) of tryptophan in rHDL was red-shifted from 339 nm to 345 nm for (1:0) and (1:2) of apoA-I:SAA, respectively, indicating that the addition of SAA to rHDL destabilized the secondary structure of apoA-I. Upon denaturation by urea treatment from 0 M to 8 M, SAA showed only a 3 nm red-shift in WMF, while apoA-I showed a 16 nm red-shift in WMF, indicating that SAA is resistant to denaturation and apoA-I had higher conformational flexibility than SAA. The glycation reaction of apoA-I in the presence of fructose was accelerated up to 1.8-fold by adding SAA in a dose-dependent manner than that of apoA-I alone. In conclusion, the incorporation of SAA in rHDL impaired the structural stability of apoA-I and exacerbated glycation of HDL and apoA-I.

## 1. Introduction

Human serum amyloid A (SAA) is an acute-phase reactant protein that is related to cardiovascular disease (CVD), whose levels increase due to acute infection, persistent inflammation, and tissue damage in the blood [1]. Human serum amyloid A1 (SAA1), which consists of 104 amino acids, was identified in the amyloid deposits in tissues of patients with chronic or recurrent inflammation [2,3]. An elevated serum SAA level is a well-known biomarker of atherosclerotic risk [4] and a predictor of future cardiovascular events [5]. SAA is an exchangeable apolipoprotein associated with high-density lipoprotein (HDL) in the plasma that is secreted during the acute phase of inflammation and is involved in the transport of cholesterol to the liver for excretion as bile [6]. Although SAA is a precursor protein in inflammation-associated amyloid A, human SAA has many functions, including amyloidogenesis, remodeling of HDL, tumor pathogenesis, anti-bacterial functions, and the regulation of inflammatory response [7,8].

Circulating SAA plays a critical role in various inflammatory diseases, including sepsis, atherosclerosis, and rheumatoid arthritis [9]. In sepsis, patients in the critical phase showed that the HDL-C level was decreased to approximately 14 mg/dL, while SAA was elevated sharply around 2827 μg/mL, while the control group showed a normal level of HDL-C (~39 mg/dL) and 269 μg/mL of SAA [10]. SAA is expressed almost exclusively in the liver and released into the bloodstream. Newly synthesized SAA displaces apolipoprotein A-I (apoA-I) and becomes an apolipoprotein of HDL [11]. SAA-enriched HDL is prone to becoming dysfunctional HDL and releasing lipid-free apoA-I [11], which helps generate poor quality HDL. High SAA and low HDL-C levels are related to elevated interleukin (IL)-6 and other cytokines, namely the “cytokine storm” and increased hepatic production of SAA during inflammation.

SAA, which is expressed almost exclusively in the liver, triggers the production of the proinflammatory cytokines, such as IL-6, through mitogen-activated protein kinases (MAPK), p38, and nuclear factor-kappa B [12]. In the blood, most liver-derived SAA is found in HDL because SAA can become the major apolipoprotein on HDL by replacing apoA-I [13]. Serum SAA is an exchangeable apolipoprotein between HDL and low-density lipoproteins (LDL)/very low-density lipoproteins (VLDL) through elevated cholesteryl ester transfer protein (CETP) activity [14]. The binding of lipoproteins to proteoglycans increases with increasing SAA content in lipoprotein, which can accelerate atherogenesis [14]. Although the enrichment of SAA in HDL is linked to the degradation of HDL and proinflammatory change in HDL, there is limited information about the interaction of SAA and apoA-I in the lipid-free and lipid-bound state.

HDL containing apoA-I (apoA-I-HDL) exerts antioxidant, anti-inflammatory, and anti-infective activity [15]. Regarding acute viral infections, such as coronavirus disease 2019 (COVID-19), a lower HDL quantity is associated with high sensitivity to COVID-19 [16] and a high risk of death [17]. HDL functionality, particularly the glycation extent of apoA-I and paraoxonase (PON-1) activity, might be important for suppressing a severe acute respiratory syndrome coronavirus-2 (SARS-CoV-2) infection [18,19]. The elevation of SAA is a biomarker of severe COVID-19 and poor prognosis [20]. Hospitalized COVID-19 patients showed 15-fold higher SAA content in HDL proteome than non-hospitalized patients [21].

Many studies have focused on the physiological effects of SAA in the HDL state. On the other hand, although a few studies compared the effect of SAA on HDL quality [7,10,11,14], there is little information on the change in the structural–functional correlation of apoA-I in reconstituted HDL (rHDL) in the absence or presence of SAA. Therefore, it is necessary to investigate the interaction between apoA-I and SAA in lipid free-state and the change in apoA-I structure in SAA-enriched HDL. In the current study, rHDLs containing apoA-I and SAA with different molar ratios were synthesized to elucidate the physiological effects of SAA in rHDL. The structural features of rHDL were characterized by the change in glycation susceptibility and secondary structure with increasing SAA content.

## 2. Results

### 2.1. Purification and Characterization of SAA1

**Figure 1:**
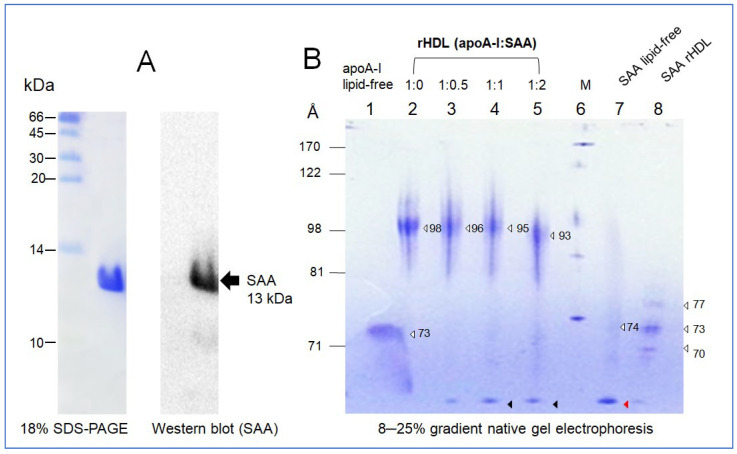
Electrophoretic patterns of purified serum amyloid A (SAA), immunodetection, and reconstituted HDL containing apoA-I and SAA. (**A**) SDS-PAGE analysis and immunodetection with anti-SAA polyclonal antibody (SC20651, Santa Cruz Biotech, CA). (**B**) Non-denaturing 8–25% gradient gel electrophoresis of apoA-I and SAA in the lipid-free and lipid-bound state. The white arrowhead symbol indicates major band of each lane. Black arrowhead indicates displaced band from each rHDL. Red arrowhead indicates aggregated band of SAA.

The calculated molecular weight of the purified SAA was 12,879 Da and isoelectric point (pI) of 6.61, with a molar extinction coefficient at 280 nm (ε_280_) of 23,950 M^−1^∙cm^−1^ using Protean analysis software version 5.0.7 of DNASTAR (Madison, WI, USA). Purified SAA1 (around 13 kDa), at least 98% purity based on SDS-PAGE (Figure 1A), showed an identical protein sequence to P0DJI8 (SAA1_Human) of the UniProtKB-sequence, starting with NH_2_-MRSFFSFLGEAFDGAR- from N-terminal protein sequencing. Western blotting and immunodetection with a SAA1-specific antibody (SC20651, Santa Cruz Biotech, Santa Cruz, CA, USA) showed that the 13 kDa protein is SAA1 (Figure 1A). SAA1 consisted of 113 amino acids, including three tryptophan (Trp) at the 18th, 53rd, and 85th positions (4.3% by weight) and five tyrosine (Tyr, 6.3% by weight) without cysteine (Cys) and threonine (Thr).

### 2.2. Synthesis of rHDL with SAA1 and ApoA-I

In a nondenaturing state, 8–25% gradient native electrophoresis showed that lipid-free SAA1 had a smeared band pattern from 64 to 74 Å with an aggregated band at the bottom of the gel, as indicated by the red arrowhead (Figure 1B), while lipid-free apoA-I showed a broad band pattern at 73 Å without aggregation at the bottom.

In a lipid-bound state, synthesized as an rHDL, apoA-I-rHDL showed a distinct band with a 98 Å particle size without an aggregated band at the bottom. With an increasing amount of SAA in the rHDL, the particle size decreased gradually from 96 Å to 93 Å at apoA-I:SAA (1:0.5) to (1:2), respectively. During rHDL synthesis, the rHDL solution showed more turbidity with an increased SAA content (data not shown), indicating that there might be a displacement of apoA-I. The displaced bands were detected at the bottom of the native gel, as indicated by the black arrowhead (Figure 1B). The rHDL-(1:0.5) (lane 3) showed the smallest displaced band, but rHDL-(1:0) showed no displaced band in the bottom of the gel (lane 2, Figure 1B). The black arrowhead showed no difference in the displaced band size between the rHDL-(1:1) and rHDL-(1:2). These results suggest that the displacement occurred dose-dependently on SAA concentration up to a 1:1 molar ratio. Interestingly, SAA-rHDL showed three small particles, 77, 73, and 70 Å, without aggregated bands, indicating that SAA alone could be synthesized as HDL, similar to apoA-I, even though the particle size was much smaller.

### 2.3. Phospholipid-Binding Assay

**Figure 2:**
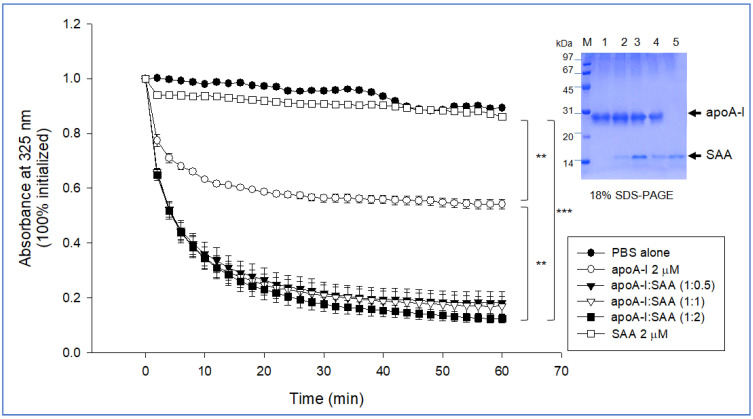
Phospholipid binding assay to compare the kinetics of apoA-I and serum amyloid A (SAA) with DMPC multilamellar liposomes. The absorbance at 325 nm was monitored at 24.5 °C at 2 min intervals. ** *p* < 0.01 between SAA alone and apoA-I alone; ** *p* < 0.01 between apoA-I alone and apoA-I:SAA (1:2); ***, *p* < 0.001 between SAA alone and apoA-I:SAA (1:2). The inset photograph shows electrophoretic patterns of the DMPC multilamellar liposomes containing apoA-I, SAA, and their mixture after a 60 min reaction (18% SDS-PAGE). Lane M, molecular weight marker (Bio-Rad low range); lane 1, apoA-I alone (final 2 μM); lane 2, apoA-I:SAA (1:0.5, molar ratio); lane 3, apoA-I:SAA (1:1, molar ratio); lane 4, apoA-I:SAA (1:2, molar ratio); lane 5, SAA alone (2 μM).

A dimyristoyl phosphatidylcholine (DMPC) binding assay showed that 46% of DMPC was cleared by apoA-I during 60 min. In contrast, SAA showed a loss of clearance, as shown in Figure 2, indicating that SAA alone could not bind with DMPC. This result revealed a remarkable difference in phospholipid binding ability between apoA-I and SAA. Interestingly and unexpectedly, the addition of SAA to apoA-I increased the DMPC clearance activity in a dose-dependent manner; up to 82%, 83%, and 88% of DMPC was cleared at apoA-I:SAA (1:0.5), (1:1), and (1:2), respectively. For 60 min, a mixture of apoA-I:SAA (1:2) showed 1.8-fold higher phospholipid-binding ability than apoA-I alone. This result suggests that a putative interaction between the helix domain of apoA-I and SAA, resulting in higher phospholipid-binding ability, even though SAA alone could not bind with the DMPC.

### 2.4. Change in Secondary Structure and Trp Movement

Circular dichroism (CD) revealed 53% and 36% of α-helicity in native apoA-I and SAA, respectively, in the lipid-free state. In the lipid-bound state, apoA-I-rHDL showed an up to 73% increase in α-helicity with a mainly 98 Å particle size, while SAA-rHDL still showed 36% with a 77, 73, and 70 Å particle size. This result suggests that apoA-I could expand the α-helicity around 20% upon phospholipid binding, but SAA could not expand the α-helicity.

Fluorospectroscopic observation showed that lipid-free apoA-I exhibited a wavelength maximum fluorescence (WMF) of 342 nm, while lipid-free SAA showed a WMF of 352 nm, suggesting that Trp in SAA was 10 nm more exposed to water-phase than apoA-I. In the lipid-bound state, apoA-I-rHDL showed an 11 nm blue shift WMF than SAA-rHDL, indicating that Trp in apoA-I moved to a more hydrophobic phase with a 3 nm blue shift in the rHDL state, while SAA showed only a 2 nm blue shift.

**Figure 3:**
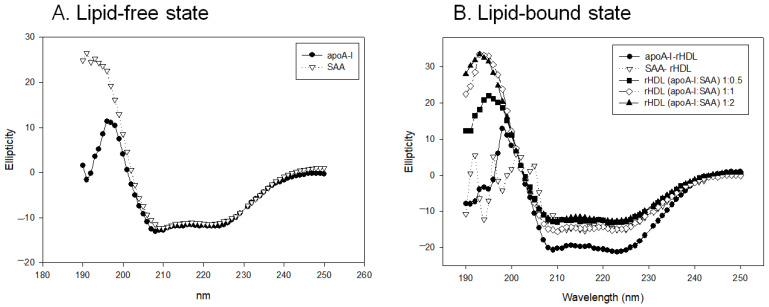
Circular dichroism spectra of apoA-I and serum amyloid A (SAA) in the lipid-free state (**A**) and lipid-bound state (**B**) as reconstituted high-density lipoproteins (rHDL) with different molar ratios of apoA-I:SAA.

Circular dichroism spectroscopy showed that apoA-I and SAA in the lipid-free state had similar ellipticity and a typical spectrum of alpha-helical proteins with two minima at 208 nm and 222 nm, as shown in Figure 3A. SAA exhibited maximal ellipticity around +26.956 at 191 nm, while apoA-I showed smaller maximal ellipticity at 195 nm, then decreased back to approximately −0.223 at 191 nm.

In the lipid-bound state, apoA-I-rHDL exhibited 73% α-helicity and larger ellipticity with two minima at 209 (−18.493) and 222 nm (−18.887), while SAA-rHDL showed 36% α-helicity with two minima at 210 nm (−9.223) and 222 nm (−9.047), as shown in Figure 3B. On the other hand, increasing the SAA content in apoA-I-rHDL induced a 54%, 49%, and 40% decrease in α-helicity for apoA-I:SAA ratios of 1:0.5, 1:1, and 1:2, respectively.

### 2.5. SAA Accelerated Isothermal Denaturation

**Figure 4:**
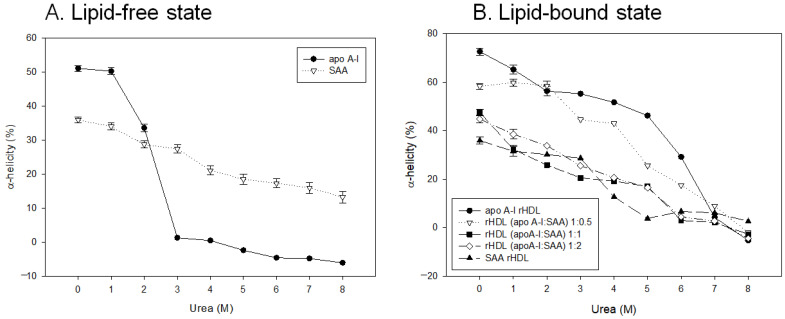
Isothermal denaturation of apoA-I and serum amyloid A (SAA) in the lipid-free state (**A**) and the lipid-bound state (**B**) as reconstituted high-density lipoproteins (rHDL) with different molar ratios of apoA-I:SAA. With increasing urea treatment, the change in α-helicity was compared by circular dichroism spectroscopy.

As shown in Figure 4A, lipid-free apoA-I exhibited resistance to 1 M urea addition with 51% α-helicity. Increasing the urea concentration (final 2–3 M) in the lipid-free state caused the rapid denaturation of apoA-I to exhibit a loss of secondary structure; 33.5% and 1.3% of α-helicity were detected at the addition of 2 M and 3 M urea, respectively. On the other hand, lipid-free SAA was relatively more resistant to the addition of urea from 1 M to 8 M; 34.1% and 13.2% α-helicity, respectively, were maintained from an initial 36% α-helicity at 0 M urea.

In the lipid-bound state, as shown in Figure 4B, the addition of SAA to rHDL caused the loss of α-helicity dependent on the SAA content, around 58%, 47%, and 44% for 1:0.5, 1:1, and 1:2 apoA-I:SAA in rHDL, respectively, under 0 M of urea, indicating that a putative interaction of SAA impaired the α-helicity of apoA-I via the displacement of apoA-I from the rHDL. Among rHDL, until 5M urea, apoA-I-rHDL showed the slowest loss of α-helicity, while SAA-rHDL showed the fastest speed of denaturation. In the presence of 5 M urea, rHDL containing apoA-I:SAA (1:0) exhibited 46% α-helicity, while (1:0.5), (1:1), (1:2), and (0:1) showed 25%, 17%, 16%, and 4% α-helicity, respectively. These results suggest that the conformational stability of apoA-I was disrupted by adding SAA to rHDL in a dose-dependent manner to facilitate more denaturation.

**Figure 5:**
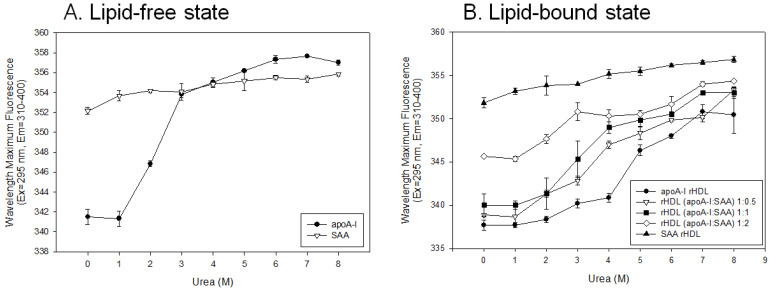
Isothermal denaturation of apoA-I and serum amyloid A (SAA) in the lipid-free state (**A**) and lipid-bound state (**B**) as reconstituted high-density lipoproteins (rHDL) with different molar ratios of apoA-I:SAA. With increasing urea treatment, change in Trp exposure was compared by fluorospectroscopy (Ex = 295 nm, Em = 310–400 nm) as wavelength maximum fluorescence (WMF).

As shown in Figure 5A, lipid-free apoA-I showed the same WMF around 341 nm in the presence of 0 M and 1 M urea, suggesting that apoA-I was resistant to denaturation against 1 M urea. On the other hand, WMF was red-shifted 5 nm and 13 nm by a 2 M and 4 M urea treatment, respectively, indicating that denaturation occurred by exposing Trp to the water phase. Denaturation was saturated at 5 M urea with 357 nm of WMF, apoA-I showed a 16 nm red-shifted WMF with a urea treatment from 0 M to 8 M. By contrast, SAA showed only a 4 nm red-shifted WMF (352 to 356 nm) with a urea treatment from 0 M to 8 M, indicating that the Trp was already more exposed to the water phase than that of apoA-I. In the lipid-free state, apoA-I had a more highly ordered secondary structure with a higher level of α-helicity with an 11 nm blue-shifted WMF than SAA.

In the lipid-bound state, adding SAA to apoA-I-rHDL caused an up to 8 nm larger red-shift in WMF in a dose-dependent manner from 337 nm, 339 nm, 341 nm, and 345 nm at 1:0, 1:0.5, 1:1, and 1:2 of apoA-I:SAA, respectively. In the presence of urea, the higher SAA content in rHDL was associated with the smaller red-shift in WMF, 15 nm, 13 nm, and 9 nm difference between 0 M and 8 M urea for an apoA-I:SAA ratio of 1:0.5, 1:1, and 1:2, respectively. Interestingly, a higher SAA content in rHDL was associated with a lower extent of denaturation. This can be explained by the more disordered structure in SAA, leaving less room for a change in α-helicity and WMF upon the denaturation process by urea.

### 2.6. SAA Accelerated the Glycation of ApoA-I

**Figure 6:**
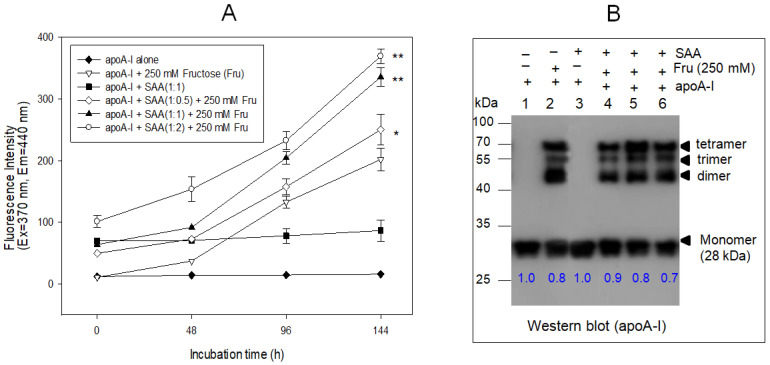
Glycation of apoA-I and SAA by fructose (Fru, final 250 mM) treatment. (**A**) Fluorospectroscopic determination of advanced glycated end product (Ex = 370 nm, Em = 440 nm). * *p* < 0.05 versus apoA-I + Fru; ** *p* < 0.01 versus apoA-I + Fru. (**B**) Immunodetection of native or glycated apoA-I, SAA, and their mixture. Lane 1, apoA-I alone; lane 2, apoA-I + Fru; lane 3, apoA-I + SAA; lane 4, apoA-I + SAA (1:0.5) + Fru; lane 5, apoA-I + SAA (1:1) + Fru; lane 6, apoA-I + SAA (1:2) + Fru. The blue numbers in the photograph indicate the band intensities (BI) of each apoA-I band. The BI was compared by band scanning with Chemi-Doc^®^ XR (Bio-Rad, Hercules, CA, USA) using Quantity One software (version 4.5.2) from three independent blots.

As shown in Figure 6A, in the absence of fructose, the addition of SAA to apoA-I with a 1:1 molar ratio did not cause the glycation and multimerization of apoA-I (lane 3, Figure 6B), similar to apoA-I alone (lane 1). On the other hand, increasing the SAA content in the presence of fructose accelerated glycation by up to 1.2-fold, 1.6-fold, and 1.8-fold at 1:0.5, 1:1, and 1:2, respectively, compared to apoA-I alone (1:0) (Figure 6A). Immunodetection (Figure 6B) using the apoA-I antibody showed that the monomeric band of apoA-I decreased gradually to 30% with increasing SAA content up to 2 μM in the presence of fructose (lane 6, Figure 6B). These results show that the glycation of apoA-I and the disappearance of monomeric apoA-I with the multimerization of apoA-I were accelerated more by the presence of SAA in a dose-dependent manner.

### 2.7. SAA Accelerated the Glycation of HDL

**Figure 7:**
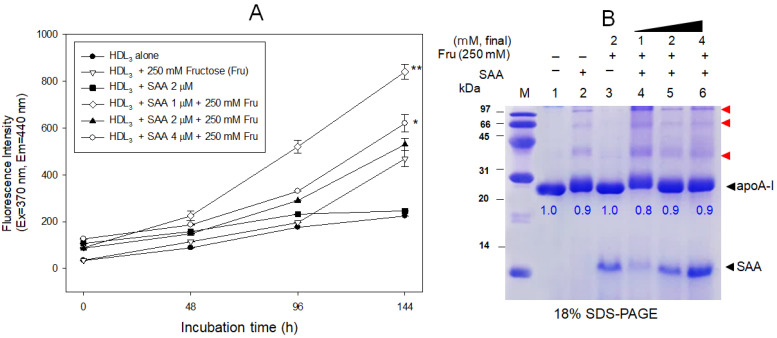
Increase in the extent of glycation in HDL_3_ by co-treatment of serum amyloid A and fructose (Fru, final 250 mM). (**A**) Fluorospectroscopic determination of the advanced glycated end product (Ex = 370 nm, Em = 440 nm) in the HDL_3_. *, *p* < 0.05 versus HDL_3_ + Fru; **, *p* < 0.01 versus HDL_3_ + Fru. (**B**) Electrophoretic patterns of glycated HDL_3_ with visualization of Coomassie Blue staining (18% SDS-PAGE). The red arrow indicates a multimerized band of apoA-I due to glycation. Lane 1, HDL_3_ alone; lane 2, HDL_3_ + Fru; lane 3, HDL_3_ + SAA (2 μM); lane 4, HDL_3_ + SAA (1 μM) + Fru; lane 5, HDL_3_ + SAA (2 μM) + Fru; lane 6, HDL_3_ + SAA (4 μM) + Fru. The blue numbers in the photograph indicate the band intensities (BI) of each apoA-I band. The BI was compared by band scanning with Chemi-Doc^®^ XR (Bio-Rad, Hercules, CA, USA) using Quantity One software (version 4.5.2) from three independent SDS-PAGE.

During 144 h incubation, in the absence of fructose, the addition of SAA (final 2 μM) did not cause the glycation of HDL_3_, similar to a low FI level of HDL_3_ alone (Figure 7A), and no multimerized apoA-I band in HDL_3_ alone (lane 1 and 3, Figure 7B). Fructose (final 250 mM)-treated HDL_3_ showed a two-fold higher glycated fluorescence intensity (FI) than HDL_3_ alone. On the other hand, the co-treatment of SAA (final 1 μM) and fructose resulted in a 3.8 times higher glycation than HDL_3_ alone, indicating a synergistic effect in causing glycation, but there was no dose-dependency of SAA.

As shown in Figure 7B, electrophoresis showed that glycated HDL produced a multimerized band of apoA-I up to the tetramer (lane 2), while HDL_3_ alone and SAA alone did not (lane 3). The co-treatment of SAA (final 1 μM) and fructose (final 250 mM) resulted in the disappearance of the monomeric apoA-I band up to 20%, and the strongest multimerized band of apoA-I with slightly more smear and a shifted-up band position (lane 4). Despite the lack of dose-dependency in the 2 μM of SAA (lane 5) and 4 μM of SAA (lane 6) treatment, the extent of glycation and multimerization of apoA-I was accelerated by the co-presence of SAA.

## 3. Discussion

Human SAA is a family of apolipoproteins that can bind to HDL during inflammation and induce the production of dysfunctional HDL by replacing apoA-I and impairing structural stability [22,23]. The elevated serum SAA in response to acute and chronic inflammation is linked to the progression of autoimmune diseases and tumor neoplasia. Because SAA is poorly soluble in aqueous solution, SAA in the blood should be partitioned into HDL via putative protein–protein and protein–lipid interactions. On the other hand, the molecular mechanism of the protein interactions between apoA-I and SAA has not been investigated thoroughly in the lipid-free and lipid-bound states. The current study compared the structural changes in HDL and apoA-I in the presence or absence of SAA. In addition to the interaction between apoA-I and SAA, the physiological effect of SAA in the lipid-free and lipid-bound states were evaluated in terms of in vitro glycation by a fructose treatment. This study is the first to compare the HDL and apoA-I quality in the absence or presence of SAA regarding the change in structural stability and glycation susceptibility.

The addition of purified SAA (Figure 1A) to the synthesis of rHDL containing apoA-I resulted in the production of smaller particles with increasing SAA content (Figure 1B) from 98 Å to 93 Å with a 6 nm red-shift in WMF and a decrease in α-helicity (Table 1). This result agrees with a previous report that the expression and characterization of the SAA isoforms in rHDL showed a similar particle size and shape [24]. Upon binding of a phospholipid, human SAA1.1 showed a 9 nm blue shift from 344 nm in the lipid-free state to 335 nm in the lipid-bound state. The α-helicity was also increased from 16% in the lipid-free state to 39% in the lipid-bound state. On the other hand, the previous report did not compare the change in α-helicity rHDL containing apoA-I and SAA in the previous report [24]. Similarly, a recent report showed that the incorporation of Aβ also caused a decrease in rHDL particle size to approximately 93 Å and a decrease in α-helicity with 3 nm red-shift at apoA-I:Aβ (1:2, molar ratio) [25]. Interestingly, there was no difference in the α-helicity content in SAA, approximately 36%, between the lipid-free and lipid-bound states. These results correlated well with the fact that SAA showed almost no phospholipid-binding ability (Figure 2) and showed only a 3 nm change in WMF during denaturation (Figure 5).

In contrast to the current results, treatment of purified HDL (0.5 mg/mL) with SAA increased the HDL size with increasing SAA content up to a 4:1 molar ratio via apoA-I displacement [26]. The difference might have originated from the different behavior of SAA in the synthesized rHDL and purified HDL. Although the displacement of apoA-I is concentration dependent on SAA, 200 μg to 1 mg is necessary to displace apoA-I in HDL [14]. On the other hand, another study reported that a low concentration of SAA in HDL, 1:0.5 molar ratio of apoA-I:SAA, also caused the displacement of apoA-I [26]. Furthermore, in rHDL synthesis with DMPC, low content of SAA (0.025 mg/mL) caused the displacement of apoA-I in the rHDL [24]. The current results also showed low contents of SAA in rHDL, 1:0.5 and 1:1 molar ratios of apoA-I:SAA. Overall, these results suggest that the displacement of apo A-I might differ depending on the SAA concentration in native HDL and rHDL.

There was a difference between the exogenous treatment of HDL with SAA and the endogenous incorporation of SAA by rHDL because purified HDL contained TG, while rHDL had no TG. The increase in TG in the HDL core augmented apolipoprotein dissociation from the surface. Therefore, TG-lowering therapies might be a promising strategy for alleviating autoimmune and amyloid diseases. Indeed, female patients with RA showed an approximately two-fold higher TG level in the serum and HDL than the control with severe amyloidogenesis in HDL [27].

Lipid-free SAA alone (lane 7, Figure 1B) showed the adequate ability of rHDL formation with POPC and cholesterol (lane 8, Figure 1B). The addition of SAA enhanced the phospholipid (DMPC) binding ability of apoA-I, but SAA alone did not bind with DMPC during 60 min and 120 min incubation (Figure 2). Interestingly, it has been reported that SAA1 could solubilize the DMPC solution at higher protein concentrations, e.g., 2;1 (*w*/*w*, SAA/DMPC), for 10 min of incubation from the light scattering intensity at 600 nm [24]. The current result suggests that the putative synergistic interaction of apoA-I and SAA increases the phospholipid-binding ability. This result may explain why the most circulating SAA is bound to the surface of HDL through the higher affinity of phospholipid and SAA into HDL, even though SAA is not incorporated into HDL during HDL biogenesis [28]. An injection of LPS caused a remarkable elevation of SAA in the blood, and most of the SAA eluted in the HDL particle in the lipopolysaccharide injected mice [29].

In the presence of fructose, glycation of lipid-free apoA-I and its multimerization was elevated by increasing the amount of SAA (Figure 6). On the other hand, the glycation of HDL_3_ reached the highest level by adding low levels of SAA (final 1 μM), as shown in Figure 7. Interestingly, the lowest content of SAA (1 μM) induced the strongest glycation extent (Figure 7A) and multimerization of apoA-I (Figure 7B). It is unclear why the extent of apoA-I glycation showed a dose-dependency of SAA while HDL_3_ did not. These results suggest that the intercalation of the SAA domain into the apoA-I helix domain in a lipid-free state occurred more easily than that of mature HDL, which was filled with more proteins and lipids.

The molecular structure of lipid-free SAA showed that residues 1–88 formed a unique Y-shaped 4-helix bundle with a polar C-terminal tail (residues 89–104) containing a short 3/10 helix [30]. The C-terminal region polar segment (residues 70–104), which is the amphipathic helix domain for lipid binding, recruits receptors and functional ligands for lipoproteins. CD analysis showed no difference in α-helicity in SAA between the lipid-free and lipid-bound states, approximately 36% (Table 1). Hence, there might be no conformational adaptability of the helix domain upon lipid binding. Furthermore, after denaturation by a urea treatment, SAA showed a much slower denaturation speed than apoA-I and a gradual decrease in α-helicity (Figure 4). At 8 M urea, SAA still exhibited 13% α-helicity, whereas apoA-I showed complete loss of α-helicity, suggesting that SAA has stronger structural stability against the denaturation process.

SAA had five helix domains and three Trp at the 18th Trp in helix 1 (1–27 amino acid), 53rd Trp in helix 3 (51–69 amino acid), and 85th Trp in helix 4 (73–88). Upon lipid binding, the movement of the helix domain and Trp exposure did not occur from WMF analysis, only a 2 nm blue shift in WMF in the lipid-bound state (350 nm) from the lipid-free state (352 nm). The structural rigidity of SAA might contribute to the intercalation of the apoA-I helix domain to displace HDL.

The synergistic phospholipid-binding effect of SAA and apoA-I was unique because Aβ with apoA-I showed the complete loss of the phospholipid binding ability [25], suggesting a different protein–protein interaction between the apoA-I helix domain and either Aβ or SAA. Lipid-free apoA-I is more labile to proteolysis or aggregation than lipid-bound apoA-I [31]. Murine SAA purified by ultracentrifugation from HDL also showed a smaller α-helicity of approximately 32% in 25 mM Tris-HCl/100 mM NaCl at pH8.0 [32].

Among the apolipoprotein family, the co-presence of α-synuclein (α-syn) exhibited inhibitory activity against fructose-mediated glycation and cupric ion-mediated LDL oxidation [33]. Although both α-syn and SAA have a similar molecular weight and the loss tendency of the phospholipid-binding activity, the physiological activities reveal differences in the inhibition of glycation and oxidation. The amyloidogenesis of apoA-I was accelerated by the co-presence of SAA with the multimerization of apoA-I, while α-syn inhibited the multimerization of apoA-I. Therefore, a future study should compare the different roles of SAA and α-syn in amyloidogenesis in the blood and brain. SAA-enriched HDL is prone to be dysfunctional HDL, which has a smaller particle size, with a concomitant displacement of apoA-I and an increase in TG content in HDL [34].

The limitations of this study were the synthesis of rHDL and the use of lipid-free SAA. The rHDL is not equivalent to circulating HDL in the acute phase, which is enriched with many other apolipoproteins, such as apoA-II and apoC-III, antioxidant enzymes, and many lipid species. Because most circulating SAA is not in the lipid-free state, the behavior of SAA in a mixture of apoA-I in the lipid-free state might differ from that of lipid-bound SAA in the acute phase. On the other hand, this characterization of SAA and apoA-I in the lipid-free state can also provide useful information for protein–protein domain interactions between the two exchangeable proteins. The putative interactions of SAA and apoA-I in the lipid-bound state were characterized using rHDL synthesis. Although it was difficult to determine the exact percentage of SAA-enriched HDL and SAA-only HDL in total HDL populations, SAA binds predominantly with HDL [11,14,35]. In vitro incubations of SAA and HDL up to a 3.0 ratio of apo-SAA/HDL_3_ resulted in a 9.7 ratio of apo-SAA/apoA-I in the recovered HDL with 80% SAA in the total proteins [35], suggesting that apoA-I was displaced in the HDL particle. Despite the difficulty in determining the SAA content in HDL, future studies will attempt to measure the exact percentage of SAA-enriched HDL and SAA-only HDL in the acute phase of the HDL population.

Future studies will compare the morphological or mechanical data detected by atomic force microscopy (AFM) to obtain the molecular details of the interaction between HDL particles and SAA. AFM is a useful tool for evaluating the biomechanical properties and morphological changes in HDL at a nanometer resolution, as described previously [36,37], and measuring rHDL containing apoA-I and SAA and lipid-free state apoA-I.

## 4. Materials and Methods

### 4.1. Materials

The cloned gene of human serum amyloid A (SAA1, Cat# hMU009432) was obtained from the Human Gene Bank of Korea (Daejeon, Korea). The pET30a(+) expression vector and *E. coli* BL21 (DE3) were purchased from Novagen (Madison, WI, USA). The restriction enzymes were acquired from New England BioLabs (Beverly, MA, USA). Palmitoyloleoyl phosphatidylcholine (POPC, #850457P), 1,2-dimyristoyl-sn-glycero-3-phosphocholine (DMPC, #850345), and cholesterol (ovine, #700000P) were supplied by Avanti Polar Lipids (Alabaster, AL, USA). Sodium cholate (#C1254) was purchased from Sigma (St Louis, MO, USA).

### 4.2. Expression and Purification of SAA1

The human serum amyloid A1 (SAA1) gene was cloned using a polymerase chain reaction (PCR) with the forward primer, 5′-ACCATGGTACATATGCGAAGCTTCTTTTCGTTC-3′, and reverse primer, 5′-ATGGTACTCGAGGTATTTCTCAGGCAGGC-3′, to generate *Nde I* and *Xho I* sites, amplify the PCR product and construct the expression vector.

The subcloned cDNA was inserted into the pET30a expression vector to be verified by DNA sequencing using a Sequentator (ABI7500, ABI, Foster City, CA, USA). The expressed polypeptides consisted of 113 amino acids, i.e., the mature form of 104 amino acids of SAA1 plus Met at the N-terminal and a His-tag (8 amino acids, L-E-HHHHHH) at the C-terminal. The His-tagged SAA1 gene was expressed in the *E. coli* and purified using a Ni^2+^-nitrilotriacetic acid chromatography column (Peptron, Cat#1103-3, Daejeon, Korea), as described elsewhere [38,39]. The fractions containing SAA1 were pooled and dialyzed against a buffer containing 10 mM Tris-HCl (pH 8.0) and 10% glycerol. The protein concentration was determined using the Bradford assays with bovine serum albumin (BSA) as the standard. SDS-PAGE and Coomassie Blue staining monitored the initial protein purity.

### 4.3. Protein Sequencing

Protein samples for sequencing were electrotransferred onto a PVDF membrane (Immobilon-P) using the protocol outlined by Matsudaira [40]. The NH_2_-terminal amino acid sequence of the excised band was determined using an Applied Biosystems model 491A sequencer (Foster City, CA, USA) located at the Korea Basic Research Institute (Daejeon, Korea).

### 4.4. Characterization of Secondary Structure

The average α-helix contents of the proteins in the lipid-free and lipid-bound states were measured by circular dichroism (CD) spectroscopy (J-700, Jasco, Tokyo, Japan). The spectra were obtained from 250 to 190 nm at 25 °C in a 0.1 cm path length quartz cuvette at a bandwidth of 1.0-nm, speed of 50 nm/min, and response time of 4 s. Samples of the lipid-free and lipid-bound proteins were diluted to 0.07 mg/mL to avoid self-association, whereas the lipid-bound proteins were diluted to 0.1 mg/mL. Four scans were accumulated and averaged. The α-helical content was calculated from the molar ellipticity at 222 nm [41].

### 4.5. Characterization of Trp Fluorescence

The wavelengths of maximum fluorescence (WMF) of the tryptophan (Trp) residues in apoA-I were determined from the uncorrected spectra using an LS55 spectrofluorometer (Perkin-Elmer, Norwalk, CT, USA), as described previously [42], using the WinLab software package 4.00 (Perkin–Elmer) and a 1 cm path length Suprasil quartz cuvette (Fisher Scientific, Pittsburgh, PA, USA). The samples were excited at 295 nm to avoid tyrosine fluorescence, and the emission spectra were scanned from 305 to 400 nm at room temperature [43]. For isothermal denaturation, the effects of urea addition on the secondary structures of SAA and apoA-I in a lipid-bound state were monitored by measuring the α-helicity and tryptophan movement by CD and fluorospectroscopy, as reported elsewhere [25,33].

### 4.6. Purification of Lipoproteins

LDL (1.019 < d < 1.063), HDL_2_ (1.063 < d < 1.125), and HDL_3_ (1.125 < d < 1.225) were isolated from the sera of young and healthy human males (mean age, 22 ± 2 years), who donated blood voluntarily after fasting overnight via sequential ultracentrifugation. The density was adjusted appropriately by adding NaCl and NaBr, as detailed elsewhere [44], and procedures were carried out in accordance with the standard protocols [45]. The samples were centrifuged for 24 h at 10 °C at 100,000× *g* using a Himac CP100-NX with fixed angle rotor P50AT4 (Hitachi, Tokyo, Japan) at the LipoLab of Yeungnam University. After centrifugation, each lipoprotein sample was dialyzed extensively against Tris-buffered saline (TBS; 10 mM Tris-HCl, 140 mM NaCl, and 5 mM EDTA [pH 8.0]) for 24 h to remove NaBr.

### 4.7. Purification of Human apoA-I

ApoA-I was purified from HDL by ultracentrifugation, column chromatography, and organic solvent extraction using the method described by Brewer et al. [46]. At least 95% protein purity was confirmed by SDS-PAGE.

### 4.8. Synthesis of Reconstituted HDL

Reconstituted HDL (rHDL) was prepared using the sodium cholate dialysis method [47] at an initial molar ratio of 95:5:1:0, 95:5:1:0.5, 95:5:1:1, and 95:5:1:2 for POPC:cholesterol:apoA-I:SAA, respectively. The physiological concentration of SAA, around 0.269 mg/mL [10], was mimicked using 0.23 mg and 0.46 mg of SAA for the 1:0.5 and 1:1 molar ratio, respectively, of apoA-I:SAA in 1 mL. The size and hydrodynamic diameter of the rHDL particles were determined by 8–25% native polyacrylamide gradient gel electrophoresis (PAGGE, Pharmacia Phast system) and by a comparison with standard globular proteins (GE healthcare, Cat# 17-0445-01). The relative migrations were compared via densitometric scanning analysis using a Gel Doc^®^ XR (Bio-Rad, Hercules, CA, USA) with Quantity One software, version 4.5.2.

### 4.9. Phospholipid Binding Assay

The interactions of the apoA-I and SAA with DMPC (Avanti Polar Lipids, Cat #850345) were monitored using a slight modification of the method described by Pownall et al. [48]. The DMPC to protein mass ratio was 2:1 (*w*/*w*) in a total reaction volume of 0.76 mL. The measurements were initiated after adding DMPC and monitored at 325 nm every 2 min using an Agilent 8453 UV-visible spectrophotometer (Agilent Technologies, Waldbronn, Germany) equipped with a thermocontrolled cuvette holder adjusted to 24.5 °C.

### 4.10. Glycation of apoA-I with SAA

The glycation sensitivity was compared by incubating the purified lipid-free apoA-I (final 1 mg/mL) with 250 mM D-fructose in 200 mM potassium phosphate/0.02% sodium azide buffer (pH 7.4), as reported elsewhere [49]. Because fructose-mediated apoA-I glycation resulted in the severe loss of several beneficial functions of apoA-I and HDL [50], apoA-I and HDL were incubated for up to 144 h in an atmosphere containing 5% CO_2_ at 37 °C. The extent of the advanced glycation reactions was determined by reading the fluorescence intensities at 370 nm (excitation) and 440 nm (emission), as described previously [51].

### 4.11. Western Blotting

For Figure 1A, the SAA protein (3 µg) of the lipid-free state was loaded and electrophoresed on 15% SDS-PAGE gels and detected using the anti-SAA antibody (SC20651, SC biotech, Santa Cruz, CA, USA) as the primary antibody (diluted 1:2000) and goat anti-rabbit immunoglobulin G-horseradish peroxidase (HRP) (A120-101P, Bethyl Laboratories, Montgomery, TX, USA) as the secondary antibody (diluted 1:5000). For Figure 6B, glycated apoA-I and multimerized apoA-I were detected using the anti-apoA-I antibody (Ab52945, Abcam, London, UK) as the primary antibody. The protein concentrations in the lipid-free and lipid-bound states were determined using the Bradford assay modified by Markwell et al. [52] with bovine serum albumin as a standard. Band intensities (BI) were compared by band scanning with Chemi-Doc^®^ XR (Bio-Rad, Hercules, CA, USA) using Quantity One software (version 4.5.2). The BI was compared using Student’s *t*-test on the SPSS program from three independent blots.

### 4.12. Statistical Analysis

The data in this study are expressed as the mean ± SD from at least three independent experiments with duplicate samples. Statistical analysis was performed using the SPSS software program (version 23.0; SPSS, Inc., Chicago, IL, USA). A *p*-value < 0.05 was considered significant.

## 5. Conclusions

The incorporation of SAA into rHDL with apoA-I impaired the structural stability with a smaller particle size of HDL. A mixture of apoA-I and SAA showed remarkable enhancement of phospholipid-binding ability compared to apoA-I alone or SAA alone, even though SAA showed almost no phospholipid-binding ability. Glycation of apoA-I and HDL were accelerated by the co-presence of SAA with multimerization of apoA-I.

## Figures and Tables

**Table 1:** Spectroscopic characterization of rHDL containing apoA-I and SAA in the lipid-free and lipid-bound state.

	Molar Composition (POPC:FC:apoA-I:SAA)	WMF ^a^ (nm)	α-helicity ^b^ (%)	Size (Å) ^c^
Lipid free apoA-I	-	342 ± 1	53 ± 2	-
Lipid free SAA	-	352 ± 1	36 ± 1	-
apoA-I-rHDL	95:5:1:0	339 ± 0	73 ± 4	98
SAA-rHDL	95:5:0:1	350 ± 1	36 ± 2	77, 73, 70
rHDL (apoA-I:SAA)	95:5:1:0.5	341 ± 1	54 ± 3	96
	95:5:1:1	344 ± 1	49 ± 2	95
	95:5:1:2	345 ± 1	40 ± 3	93

^a^ Determined by fluorospectroscopy (Ex = 295 nm, Em = 310–400 nm); ^b^ Determined by circular dichroism spectroscopy; ^c^ Determined by 8–25% native gradient nondenaturing gel electrophoresis and densitometry; FC, free cholesterol; POPC, palmitoyloleoyl phosphatidylcholine; rHDL, reconstituted high-density lipoproteins; SAA, serum amyloid A; WMF, wavelength of maximum fluorescence.

## Data Availability

The data used to support the findings of this study are available from the corresponding author upon reasonable request.
